# Response to “Comment on ‘Background Ionizing Radiation and the Risk of Childhood Cancer: A Census-Based Nationwide Cohort Study’”

**DOI:** 10.1289/ehp.1510111R

**Published:** 2015-08-01

**Authors:** Ben D. Spycher, Martin Röösli, Matthias Egger, Claudia E. Kuehni

**Affiliations:** 1 Institute of Social and Preventive Medicine, University of Bern, Bern, Switzerland; 2 Swiss Tropical and Public Health Institute, Basel, Switzerland; 3 University of Basel, Basel, Switzerland

Siegel and colleagues object to our use of the word “risk” on the basis that it implies a causal relationship. This is not so. In epidemiology, risk is simply the probability of developing the disease. Comparing risks across exposure strata is a natural way of assessing associations in a cohort study and does not imply causality. Our conclusions regarding causality are, in fact, very cautious.

The authors correctly point out that we are investigating low doses. The comparison they make with worldwide averages is, however, misleading. The worldwide annual dose of 2 mSv represents total background radiation and includes inhaled radon gas and ingested radionuclides. The appropriate comparison is with cosmic and terrestrial gamma radiation, which together contribute an annual average of 0.9 mSv worldwide (UNSCEAR 2000). This figure is on par with our lowest exposure category. Their comments on the use of a geographic model instead of measurements to estimate exposure reiterate limitations that we discuss in the paper.

Siegel and colleagues argue that the point estimates for the highest exposure category are unreasonably high and contradict literature showing protective effects of radiation on cancer. However, they base their argument mainly on ecological studies (Doss and Little 2014; Luckey 2008), which are prone to bias. Our study results are in line with a recent case–control study of 27,447 childhood cancer cases from the United Kingdom, which also observed a risk increase for gamma radiation (Kendall et al. 2013).

The authors suggest that other factors such as socioeconomic status and degree of urbanization are likely to explain our results. However, when we adjust for these factors, our results remain virtually unchanged. Consider the estimated response to cumulative dose, adjusted for sex and birth year (Table 4): For all childhood cancers we estimated an increase in risk per mSv cumulative dose of 2.8% (95% confidence interval [CI]: 0.8%, 4.8%) for the entire cohort and 4.0% (95% CI: 1.7%, 6.4%) for children with stable residence. After adjusting for socioeconomic status—using the Swiss neighbourhood index of socioeconomic position (Panczak et al. 2012), which is based on the education and occupation of household heads, rent, and crowding—and for degree of urbanization (urban, peri-urban, rural), the corresponding estimates were 2.9% (95% CI: 0.9%, 5.0%) and 4.0% (95% CI: 1.7%, 6.3%), respectively. The authors confuse the effects of socioeconomic status on mortality with those on cancer incidence in children. Only the latter could confound our results, but the evidence for their existence is far from conclusive (Adam et al. 2008).

The public health action proposed, i.e., the relocation of children to areas with lower radiation, is nonsensical. Childhood cancer is rare, and we are not dealing with deaths at “alarming rates.” In the whole of Switzerland, there are about 200 new cases per year, of whom more than 80% survive (SCCR 2015). Only a small proportion of the population is living in highly exposed areas. The attributable fraction, assuming a causal relationship, is therefore small. Public health action is better targeted toward modifiable environmental factors leading to larger numbers of deaths from several causes, such as exposure to radon, air pollution, and secondhand tobacco smoke.

It seems to us that the “Scientists for Accurate Radiation Information” *a priori* exclude the possibility that low-dose radiation could increase the risk of cancer. They will therefore not accept studies that challenge their foregone conclusion.
